# Implementing oral care to reduce aspiration pneumonia amongst patients with dysphagia in a South African setting

**DOI:** 10.4102/sajcd.v63i1.102

**Published:** 2016-02-16

**Authors:** Jaishika Seedat, Claire Penn

**Affiliations:** 1 Department of Speech Pathology and Audiology, University of the Witwatersrand, South Africa

## Abstract

Oral care is a crucial routine for patients with dysphagia that, when completed routinely, can prevent the development of aspiration pneumonia. There is no standardised protocol for oral care within government hospitals in South Africa. This study aimed to investigate the outcome of an oral care protocol. Participants were patients with oropharyngeal dysphagia, with either stroke or traumatic brain injury as the underlying medical pathology, and nurses. All participants were recruited from one tertiary level government hospital in Gauteng, South Africa. 139 nurses participated in the study and received training on the oral care protocol. There were two groups of participants with oropharyngeal dysphagia. Group one (study group, *n* = 23) was recruited by consecutive sampling, received regular oral care and were not restricted from drinking water; however, all other liquids were restricted. Group two (comparison group, *n* = 23) was recruited via a retrospective record review, received inconsistent oral care and were placed on thickened liquids or liquid restricted diets. Results showed that a regimen of regular oral care and free water provision when combined with dysphagia intervention did prevent aspiration pneumonia in patients with oropharyngeal dysphagia. The article highlights two key findings: that regular and routine oral care is manageable within an acute government hospital context and a strict routine of oral care can reduce aspiration pneumonia in patients with oropharyngeal dysphagia. An implication from these findings is confirmation that teamwork in acute care settings in developing contexts must be prioritised to improve dysphagia management and patient prognosis.

## Introduction

Aspiration pneumonia is serious and can be a fatal complication of oropharyngeal dysphagia (Marik & Kaplan, 2003). Aspiration of food, reflux and oral bacteria, as well as oropharyngeal dysphagia itself, contributes to aspiration pneumonia (Langmore, 1991). Martin *et al*. (1994) and Robbins *et al*. (2008) confirm a strong association between dysphagia and aspiration pneumonia due to the aspirated material being heavily colonised with bacteria. A complication of the relationship between pneumonia with aspiration and oropharyngeal dysphagia is that not all patients shown via imaging studies to have dysphagia with or without aspiration develop pneumonia (Martin *et al*., 1994). Studies investigating water protocols have queried why this would be (Becker, Tews & Lemke, 2008; Bronson-Lowe *et al*., 2008; Carlaw *et al*., 2012; Frey & Ramsberger, 2011; Garon, Engle & Ormiston, 1997; Karagiannis, Chivers & Karagiannis, 2011; Nevitt, 2010; Panther 2005, Robbins *et al*., 2008; Scott & Benjamin, 2010).

Panther (2005) introduced the Frazier Free Water Protocol, based on the premise that water has a neutral pH and, whilst providing a safe means of assessing patients with thin liquids, also allows small amounts of water that may be taken into the lungs (i.e. aspirated) to be absorbed into the body pool, with no risk of aspiration pneumonia. Despite thickened liquids being the most frequently used technique for patients with dysphagia in acute and chronic rehabilitation facilities, studies have shown that this is the consistency most patients are non-compliant with (Logemann *et al*., 2008; Robbins, Langmore, Hind & Erlichman, 2002). The lack of standardisation of viscosity with thickened textures and poor quality control thereof, unsuitability of thickening agents for patients with diabetes and a lack of empirical evidence to support the clinical perception that a thicker viscosity reduces the occurrence of aspiration support the use of water (provided the oral cavity is clean) for assessments and management of dysphagia (Frey & Ramsberger, 2011; Steele & Swigert, 2006).

Previous studies on oral care and dysphagia have focused predominantly either on patients in residential and long-term care facilities (Langmore, Skarupski, Park & Fries, 2002; Sarin, Balasubramaniam, Corcoran, Laudenbach & Stoopler, 2008) or patients in critical and intensive care settings (Grap, Munro, Ashtiani & Bryant, 2003; Rello *et al*., 2007). A paucity of evidence exists focusing specifically on oral care, dysphagia and aspiration pneumonia within a *general acute medical ward*. A general medical ward within an acute government hospital, such as in South Africa, differs from residential care facilities and high care facilities on several levels:

1. Funding. For privately funded and government subsidised facilities, resources, consumables and human resources differ. This may have implications for frequency, efficiency and willingness to engage in a routine such as oral care.
2. Patients, staff turnover, pace of work, workloads and sustainability of interventions (such as oral care) differ, which may affect implementation of procedures (Mojoyinola, 2008).
3. Training of staff. Nurses in general medical wards do not receive any specialised training on oral care protocols (Mathivha, 2002; Scribante & Bhagwanjee, 2008). This has implications for consolidation and integration of how oral care relates to dysphagia and dysphagia to aspiration pneumonia (Miller & Rubenstein, 1987).
4. The expected protocols and routines with patients differ amongst the respective contexts. Oral care is not identified as an essential routine in a general medical ward (South African Nursing Council [SANC], 2004).

Against this backdrop, the study addressed the possibility of a reduction in occurrence of aspiration pneumonia amongst patients hospitalised for oropharyngeal dysphagia in a general medical ward in an acute hospital, as has been documented internationally to be possible for patients in high care settings (Fields, 2008) and residential care facilities (Yoneyama *et al*., 2002).

## Oral care aspects for the patient with dysphagia

Colonisation of micro-flora and gram-negative bacteria is guaranteed with inactivity of the oral structures such as when there is no tooth brushing or rinsing of the mouth (Jones, 1998; Theilade, 1986). For patients with dysphagia, reduced or no activity of the oral cavity is common. Tooth brushing and general oral care are often easily neglected routines by nurses for dependent patients within general medical wards (such as for patients with dysphagia), with tooth brushing being viewed as a routine worse than nappy changing (Adams, 1996; Wårdh, Andersson & Sörensen, 1997). For patients with dysphagia there is the added complication of a resultant decrease in saliva production (Berry & Davidson, 2006). The primary functions of saliva, in addition to its lubrication function in bolus formation, are its contribution to taste sensation, its cleansing purpose as well as its role in neutralising acid produced by bacteria on tooth surfaces (Berry & Davidson, 2006; Jones, 1998).

For patients not receiving oral care, after three days of undisturbed maturation, gram-negative bacteria become more prevalent from the accumulation and formation of hundreds of species of bacteria (Marsh & Martin, 1992). Gingivitis may be evident in less than 10 days without plaque removal (Donoghue, 1987). This coupled with the highly vascular presentation of the mucosal tissue contributes to tissue destruction and consequent communication between the bacteria in the plaque and the blood stream (Kite & Pearson, 1995). During episodes of aspiration, the likelihood of bacterial transfer to the lungs is increased. A reduction in saliva production reduces the release of bacterial and fungal suppression agents thereby increasing the presence of gram-negative bacteria, which in patients with dysphagia who are aspirating, leads to aspiration pneumonia.

Oral care and hygiene is a simple and cost-effective method that could reduce the above-stated complications from both a dental and respiratory perspective. The medical and psychosocial benefits associated with regular oral care are especially significant for the patient with oropharyngeal dysphagia who is susceptible to aspiration pneumonia. Therefore, this study aimed to investigate whether it was possible to reduce the occurrence of aspiration pneumonia for patients presenting with oropharyngeal dysphagia by implementing a regular routine of oral care.

## Methodology

### Aim

To determine the outcome of an oral care regimen combined with free water provision for patients with oropharyngeal dysphagia, with a specific focus on aspiration pneumonia.

### Design

A quantitative, quasi-experimental parallel group design was necessary to determine the outcome of the regimen introduced. Concurrent change in the dependent variable, aspiration pneumonia, with introduction of the independent variables (i.e. oral care and water provision) were surmised.

### Sample

The sample consisted of two groups of participants, each with oropharyngeal dysphagia. The study group was 23 patients recruited using consecutive sampling. These participants received the scheduled oral care and were given unlimited amounts of water, which were recorded. A retrospective record review, via consecutive sampling, was used to select a pathology-matched (described below) comparison group (*n* = 23), who did not receive consistent or regular oral care. The participants in the comparison group were on liquid restricted diets; however, records confirmed that all these participants consumed various amounts of liquid, which were documented by nurses. Both groups of participants received dysphagia intervention (described below). There were equal numbers of male and female participants with dysphagia in the study. There was no significant difference in the gender composition between the participants of the study and comparison group. Participants from both groups were diagnosed with either stroke or traumatic brain injury as their primary medical diagnosis. The groups were matched for primary diagnoses as each group had similar numbers of patients with stroke and traumatic brain injury, with more stroke patients in the sample overall (stroke *n* = 32, traumatic brain injury *n* = 14; i.e. the groups were matched for pathology). All participants had a confirmed diagnosis of oropharyngeal dysphagia. No participant from either group presented with aspiration pneumonia at the initiation of dysphagia intervention (entry into the study), although signs of aspiration were observed and aspiration pneumonia developed over the course of intervention. This is detailed below. The number of nurses who participated in the study was 139. They received training on oral care by the researcher (speech-language pathologist) and each nurse participant was responsible for implementing the oral care according to a protocol (refer to Figure 2).

The researcher provided the dysphagia intervention for the participants in the study group and the resident speech-language pathologist at the site provided dysphagia intervention for the participants in the comparison group. Both had a minimum of five years’ experience working in adult neurology and adult dysphagia specifically.

#### Dysphagia intervention for the study group

**FIGURE 1:**
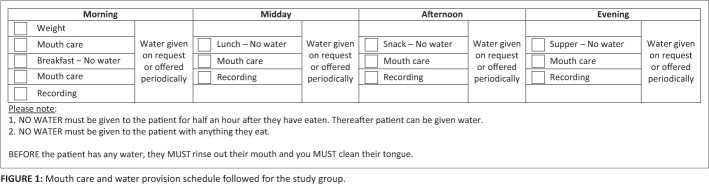
Mouth care and water provision schedule followed for the study group.

During the course of intervention, a minimum of one meal a day was observed for each participant in the study group. Participants were not restricted in their water consumption, except during meals and half an hour after a meal (refer to Figure 1). During all meals participants were repositioned in their chair or bed. Dependent participants were fed by the nurse.

**FIGURE 2:**
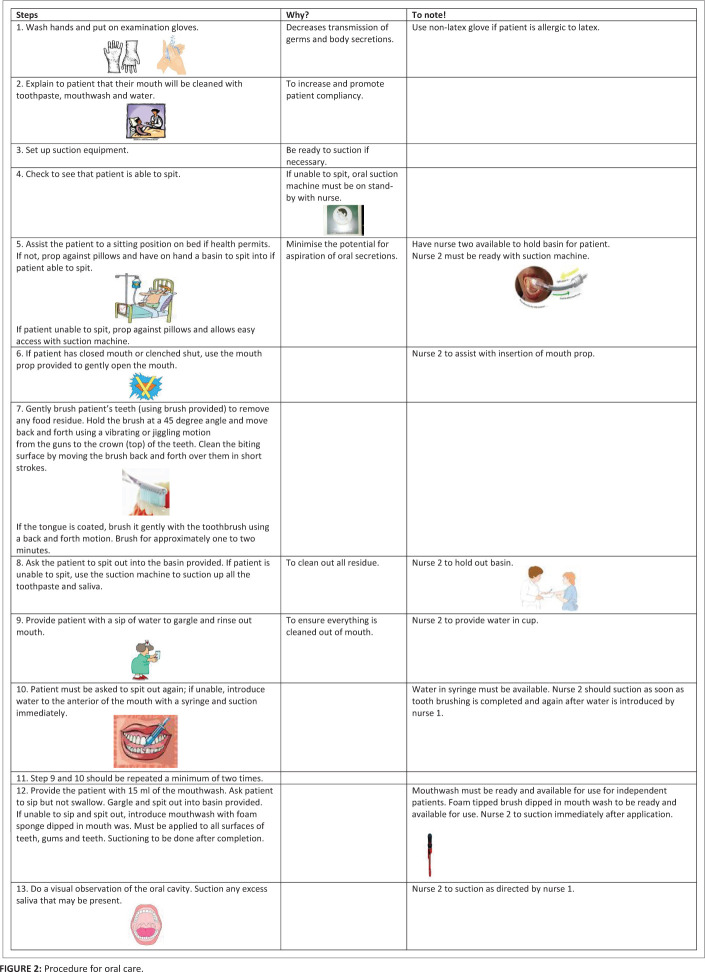
Procedure for oral care.

The following strategies were incorporated into nurse training and implemented during meals:

- Confirming correct food consistency prior to commencing with the meal.
- Pacing – allowing sufficient time for bolus preparation and swallow.
- Amount – amount offered had to align with competency of patient.
- Observation of patient between mouthfuls. Ensure clearance of oral cavity.
- Monitor patient level of wakefulness and responsiveness to the meal. Adjust accordingly.

During the course of intervention, different participants transitioned from oral to non-oral feeding and from non-oral to oral feeding based on presenting symptomatology. Participants on non-oral feeding remained within the study and continued to receive oral care and free water. Participants in the study group followed the oral care and free water regimen seen in Figure 1. Each participant received tooth brushing before breakfast, after which the participant rinsed out their mouth. This was done manually for dependent participants using a suction machine. Water was provided half an hour after breakfast and regularly until lunch time. Prior to any water consumption the participant’s mouth was rinsed. Participants or caregivers completed this routine in addition to the nurse, researcher and research assistant. The amount consumed was documented. The same protocol followed for lunch, tea and dinner. During the night, the participant did not consume any meals and received only water, if requested.

In addition to incorporating the principles of free water provision described above, dysphagia management incorporated recommendations of Cichero and Murdoch (2006), Cook and Kahrilas (1999), Langmore and Miller (1994), Logemann (1997) and Logemann (1983) with a combination of direct (involving food) and indirect (involving exercise) strategies, thermal-tactile stimulation, postural changes and behavioural techniques:

- Diet modifications: thin liquid (water), pureed foods, soft foods and solids.
- Viscosity and texture modifications excluding thickened liquid texture.
- Exercise and facilitation techniques: strengthening exercises, thermal stimulation.
- Postural adjustments: head tilt, chin tuck, head rotation, elevation.
- Compensatory: pace of feeding, amount of bolus, presentation mode.
- Manoeuvres: supraglottic swallow, super-supraglottic swallow, effortful swallow and Mendelsohn manoeuvre.
- Other: tube feeding.

The techniques and manoeuvres were used as and when necessary for each participant based on their presenting dysphagia symptoms initially and their subsequent outcome measures.

#### Dysphagia intervention for the comparison group (based on information from the retrospective record review)

Records for 13 of the 23 participants revealed that they were referred late (from between three and seven days after being admitted) and after repeated notes in the file indicating that the participant was presenting with difficulty swallowing food or drinking liquids. Referrals to the speech-language pathologist most frequently came via the dietitian. Common trends that were noted across the participant’s files in terms of the dysphagia intervention provided included:

- Participants were placed on liquid restricted diets (i.e. they were not to consume any liquids, including water).
- Mouth care was requested frequently for each of the participants.
- Mouth care was not performed by the speech-language pathologist.
- Dysphagia management for the day was often incomplete or restricted because of poor oral hygiene of the participant. This happened on several days for each of the participants.
- Specific therapeutic management strategies were vague. Notes revealed that participants were re-evaluated daily to determine if they were managing on the recommended consistency or if there was a need to modify the consistency of the diet.
- The supraglottic swallow manoeuvre and patient positioning during feeding were recommended most frequently across the group.
- None of the participants was suspected of aspiration pneumonia during the initial consultation with the speech-language pathologist. However, during the course of intervention, aspiration pneumonia was suspected with seven participants (based on signs and symptoms during the bedside evaluation, as well as temperature recordings), following which recommendations for a nasogastric tube were made. This request for each of the participants continued for five to nine days, before the feeding tube was inserted for four of the seven participants, who received oral feeds in the interim. Three participants remained on oral feeds before being discharged.
- Despite repeated requests for participants on nasogastric tube to have oral care, notes suggest that this was not done.
- When aspiration was suspected, there were recommendations for more frequent temperature monitoring. No notes of the requested additional recordings were found in the files.
- Barium swallows and chest X-rays could not be performed timeously due to malfunctioning equipment.
- There were notes by the speech-language pathologist querying that some participants were consuming liquids despite the recommendation of no thin liquids (based on information from the participants, information from the input/output chart and symptoms observed during the bedside evaluation). Requests to nurses for this to be discontinued and no liquids to be given continued over several days, after which no further mention of this was made. It is unclear how this was monitored, followed up or resolved.

It is important to note that information presented was derived from the notes in the participants’ medical files. It is likely that more dysphagia management was done with each participant than what was recorded. The limitations of the information obtained from the record review are acknowledged.

To address the aim, the following comparisons between the study group and the comparison group were documented:

*Amount of water consumed*: Would the study group participants consume more water, as there were no restrictions on drinking water except at mealtimes and half an hour after meals, than the comparison group who were on liquid restricted diets?

For the study group, water consumption was recorded by the nurse providing the water or by the participant or caregiver. For the comparison group, the information was obtained from the patient input/output chart in the medical file. For the participants in the comparison group it was significant that despite the recommendation by the speech-language pathologist that the participant be restricted from thin liquids, recordings on the input/output chart by the nurses showed intake of water, tea and coffee for each of the participants. This finding is discussed below. There was the probability that participants from both groups may have consumed more water than what was recorded. This is acknowledged as a limitation of the study.

**Oral care and aspiration pneumonia:** Would oral care reduce occurrence of aspiration pneumonia?

Although none of the participants from the study group was diagnosed with aspiration pneumonia at their entry into the study based on signs and symptoms, and observation of temperature recordings, aspiration was noted during the course of intervention. Eight participants (*n* = 3 with stroke and *n* = 5 with traumatic brain injury) from the study group exhibited irregular signs of coughing with puree consistency and episodes of throat clearing after consumption of liquid (water). Of these, two participants presented with episodes of choking at least once per meal over two days. Dysphagia management was tailored to accommodate these difficulties with the eight participants and temperature recordings showed no change over the duration of these incidents, despite signs of aspiration at the bedside. These difficulties resolved prior to discharge. This finding is discussed below.

Of the 23 participants in the comparison group, records revealed that seven participants presented with signs of aspiration and subsequently developed aspiration pneumonia during the course of management. All participants from the comparison group were discharged from hospital with recommendations to remain on thickened liquids only and for positioning during meals and pace of feeding to be monitored. The lack of detail in the notes from the medical file prevented a closer analysis of the dysphagia management; however, for the seven participants who were aspirating recommendations for insertion of a nasogastric tube were made. As noted, only four of these participants had a nasogastric tube inserted; the remaining three continued on oral feeds. There were notes by the speech-language pathologist requesting that the participants remain in hospital until the swallowing improved (or aspiration resolved); however, a shortage of beds at the site prevented longer hospitalisation for the seven participants. Five participants were discharged home, one to a nursing facility and one participant was transferred to a different acute care hospital.

The schedule for oral care has already been described under the dysphagia intervention section. It can be viewed in Figure 1. The actual oral care routine has been detailed in Figure 1. It provides a step-by-step protocol for how the oral care should be done, the structures and surfaces that need to be cleaned and the equipment and resources that are needed to achieve this, all with diagrams. A troubleshooting section is also included for common difficulties that may be encountered when completing oral care for a patient.

A limitation of the current study was the exclusion of videofluroscopy pre-intervention for each participant in the study group to confirm swallowing function. The presence of aspiration pneumonia was however monitored throughout the intervention period based on changes in patient symptomatology, patient reports and temperature monitoring. For the study group, a chest X-ray and videofluroscopy were completed at the end of intervention to essentially confirm no lung infection (aspiration pneumonia) and adequate swallowing. For the comparison group, only participants with overt symptoms of aspiration pneumonia could be referred for a barium swallow if the equipment was working at that time. The discrepancy in the use of a different imaging study for participants in each group is explained in the discussion.

### Research process and data collection

#### Ethical considerations

**FIGURE 3:**
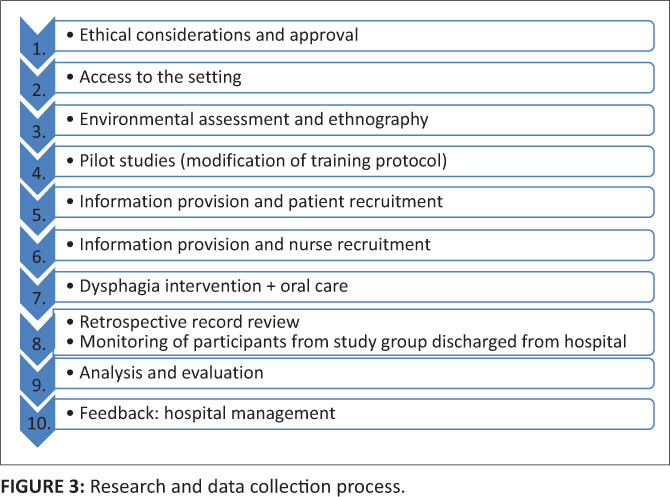
Research and data collection process.

The research process followed is shown in Figure 3. The study was conducted at an acute tertiary level public hospital in Johannesburg, Gauteng. Necessary ethical approval and consent was obtained from the University of the Witwatersrand Human and Ethics Committee (Medical) prior to commencement (M091166).

Within a larger study (Seedat, 2013) nurses were initially observed as part of a hospital ethnography. Nurse-patient interactions and nurse routines were amongst the different elements observed. These observations informed the need for, the content and length as well as means of implementing an oral care training protocol for nurses. The training protocol underwent several phases of modification until it was shown to be the most practical and efficient for nurses working within a general medical ward in an acute government hospital. The training is not described in detail within the current article, as this training process is described in an article currently in preparation.

Information included in the training with the nurses included details of when oral care needed to be implemented (Figure 1), the protocol to be followed, use of equipment and troubleshooting suggestions for non-compliant patients (Figure 1). The oral care schedule was self-devised but based on principles of the Frazier Free Water Protocol (Panther, 2005). A training manual accompanied the hands-on training on the oral care routine that nurses received. The training protocol was designed to be particularly sensitive to resource and time constraints experienced by nurses working in an acute general medical ward whilst facilitating skill refinement by providing continuing professional development certification on completion of the training.

Data for the study group was collected daily over a period of four months. The duration and composition of dysphagia intervention was participant specific and varied. To limit the potential for aspiration, the study group participants did not consume any liquids aside from water for the duration of their dysphagia intervention. This was monitored and documented by the nurse who was implementing the oral care. Cubicle nursing meant that one nurse was responsible for three to four patients during their shift. As can be seen in Figure 3, the patient with dysphagia therefore had to be identified and recruited into the study first. Thereafter the nurse responsible for that participant was recruited into the study and received the training. Each nurse was trained once. Nurses were usually allocated to the same patients they were caring for in their previous shift. Nevertheless, it was necessary for nurse training to happen daily, as nurses did rotate. For the comparison group, records for one calendar year were reviewed until the required number of participants were recruited.

During the dysphagia and oral care provision, the nurse participant recorded each implementation of oral care for the participant and documented the amount of water consumed, after each consumption. The researcher, to ensure adherence and consistency of oral care implementation and water provision, monitored both these recordings daily. The oral care protocol specified that participant’s mouths were cleaned prior to any water consumption and after each meal consumed, to prevent pooling and bacteria build-up from residue in the oral cavity, commonly seen in patients with oropharyngeal dysphagia. During the documentations, nurses commented on difficulties encountered during the oral care routine. These were addressed by the researcher and research assistant as the records were reviewed regularly throughout the day. Incomplete records could also be followed up immediately with the nurse responsible. Dysphagia intervention was provided simultaneously by the researcher. When the participant was able to safely swallow two different food consistencies based on subjective bedside evaluation, and after consultation with the attending doctor, a decision for discharge was made. Prior to discharge the participant had a videofluroscopy and chest X-ray. Following this, the participant was discharged and monitored for a period of three months thereafter by the researcher, after which time they were given an outpatient appointment or were told to contact the resident speech-language pathologist at the hospital for any changes to eating and swallowing.

For reasons pertaining to non-maleficence, it was deemed unethical to withhold oral care and water provision to patients with dysphagia; hence, a retrospective record review was instead used to recruit the comparison group participants. These participants had received the ‘standard’ dysphagia management, which included inconsistent oral care, and were ‘restricted’ from thin liquids. Similar inclusion and exclusion criteria were used to recruit the study and comparison group participants. Participants in the comparison group did not routinely undergo a barium swallow. There was no nurse training that accompanied the comparison group, as this was not a component of standard dysphagia intervention.

## Data analysis

The Statistical Analysis Software was used, incorporating non-parametric statistical procedures. The 95% confidence level was used throughout, unless otherwise specified. The Wilcoxin two-sample test, Kruskal-Wallis test and Spearman’s rank correlation were used to make within-group comparisons for the study group. Skewness of the data (large variation) prevented the use of parametric procedures, hence non-parametric statistical procedures.

## Results

### Aspiration pneumonia

**FIGURE 4:**
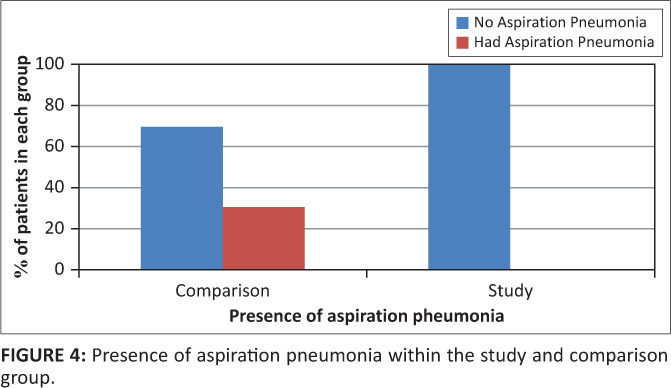
Presence of aspiration pneumonia within the study and comparison group.

Across the sample, seven participants presented with aspiration pneumonia. The Fisher’s exact test showed that there was a significant, moderate association between the occurrence of aspiration pneumonia and group: all seven were participants from the comparison group (*p* = 0.0092). This is seen in Figure 4. Four of these participants underwent a barium swallow preceded by a chest X-ray that confirmed lung infection in the absence of any other infection, which suggested aspiration pneumonia. The remaining three participants received only a chest X-ray because of malfunctioning equipment.

All 23 participants in the study group received a chest X-ray and videofluroscopy at the end of their period of dysphagia intervention. Correlating with Figure 4, none of the participants who received regular oral care presented with aspiration pneumonia at the end of their period of dysphagia intervention. Incidents of penetration and aspiration with coughing, throat clearing and choking were prevented from becoming aspiration pneumonia largely due to the strict oral care regimen and water intake preceded by mouth rinsing, monitoring of intake by the nurse and the daily communication between nurse, the speech-language pathologist and participant or caregiver.

### Amount of water consumed

Analysis was completed looking at the relationship between the amount of water consumed and the duration of intervention. Adherence to an oral care regimen, monitored dysphagia intervention and unrestricted water consumption (except at meal times) led to the following hypotheses: (1) the study group would consume more water and (2) the longer the duration of intervention (per participant), the more the water consumption.

The first hypothesis was not proven. The comparison group consumed more water than the participants in the study group did. Aligning with this unexpected finding, the result showed poor monitoring of food and liquid intake for the participants in the comparison group by nursing staff and noncompliance with recommendations of no thin liquid intake, at various levels. Notes in the participant’s files revealed poor follow-through of recommendations made by the speech-language pathologist for nurses to order specific diets for participants.

**FIGURE 5:**
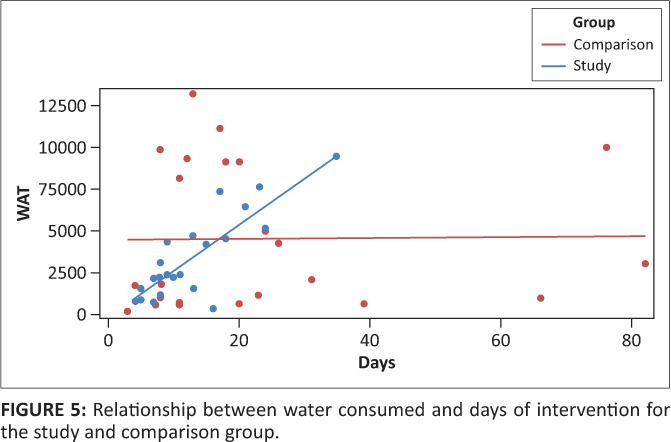
Relationship between water consumed and days of intervention for the study and comparison group.

With regard to the second hypothesis, for the study group there was indeed a significant, positive correlation between these variables (i.e. water consumption increased as the duration of intervention lengthened; correlation coefficient, *r* = 0.84; *p* < 0.0001). This is evident in Figure 5. However for the comparison group, there was no significant correlation between these variables (*r* = 0.014; *p* = 0.95), showing that the total amount of water consumed by patients did not increase with increased duration of intervention.

### Oral care

Overall, there were three meals that were not accompanied by oral care for the study group participants. Time constraints due to workload and routines considered more priority, contributed to these care sessions being incomplete (qualitative data can be viewed in Seedat, 2013). There was a positive correlation between the oral care provided and the absence of aspiration pneumonia indicated by the chest X-rays. This suggested that oral care prevented build-up of oral bacteria. Regular cleaning ensured good clearance of gram-negative bacteria amongst participants who were susceptible to aspiration. The overall result was no occurrence of aspiration pneumonia amongst any of the study group participants.

To determine if there was a significant difference between the stroke and traumatic brain injury participants for aspiration pneumonia, amount of water consumed and duration of intervention, a GLM (General Linear Model) analysis was completed for each of the listed variables. No significant differences were noted between the two underlying pathologies for any of the variables.

## Discussion

As with the findings of Yoon and Steele (2007), poor to no oral care amongst the comparison group participants culminated in seven participants acquiring aspiration pneumonia. Yoon and Steele highlight that regular oral care shows a decrease in respiratory pathogens that lead to aspiration pneumonia; hence, oral care decreases occurrence of aspiration pneumonia, as was noted amongst the participants from the study group. As was seen amongst participants from the study group, even with episodes of aspiration, if the oral cavity is free of bacteria, then no bacteria is transferred to the lungs to enable development of infection (i.e. aspiration pneumonia), a sentiment reiterated by Panther (2005).

The finding of the comparison group supports findings of Wårdh *et al*. (1997), which showed that unless monitored, oral care is a routine often disregarded in a general medical ward. Notes by the speech-language pathologist confirmed lack of tooth brushing and poor if any rinsing of the oral cavity before or after meals, which inevitably resulted in a build-up of bacteria on the surfaces of the oral cavity (Yoon & Steele, 2007). Dry and cracked lips, tongue and oral cavity immediately opens up a pathway for infection, allowing communication between the bacteria in the oral cavity and the blood stream, increasing the chances of bacterial transfer to the lungs (Kite & Pearson, 1995; Millins, Gosney, Jack, Martin & Wright, 2003). In essence the bacteria overwhelm an already impaired host-defence system, especially seen in patients with neurological impairment such as stroke (Byers & Sole, 2000). Thus, participants in the comparison group by virtue of poor oral care and oral hygiene were more susceptible to acquiring aspiration pneumonia. Findings of the study confirmed that this was the case with seven participants.

As with the study group, each participant in the comparison group received individual assessment and management strategies based on the underlying cause of the swallowing difficulty and presentation of the dysphagia. Although there was minimal detail of dysphagia management provided for the comparison group participants, it was noted that poor inter-professional communication adversely affected patient prognosis (Logemann, 1994). For instance, the repeated requests for oral care may have been alleviated with direct communication with nurses and nursing managers. A lack of accountability served to perpetuate poor follow-through of recommendations made by the speech-language pathologist. Logemann (1994) notes that successful management of the patient with dysphagia cannot be achieved by any one professional alone. A routine of oral care falls within the realm of the nurse (SANC, 2004) and, as was noted from the results of the study group participants who presented with instances of penetration and aspiration, it can minimise if not prevent occurrence of aspiration pneumonia.

A further point to elucidate the shortcoming of poor team management of dysphagia, and particularly inclusion of the patient or caregiver, was seen in the noncompliance with recommendations of thickened liquids for the comparison group participants. Colodny (2005), Rosenvinge and Starke (2005) and Logemann *et al*. (2008) have confirmed that patients are more likely to be non-compliant with recommendations made if they have no understanding of why it is necessary. Similar reasons underlie why nurses too do not enforce recommendations by allied health professionals (Begley, 2009). Improved compliance with oral care and water provision that was noted by the nurses caring for the participants in the study group was due to two primary reasons: (1) an understanding of why oral care and water provision was important for patients with dysphagia, with an understanding of how these routines needed to happen, and (2) monitoring of these routines. Hence, involving patients in decisions being made about their health and nurses of their role in patient management resulted in positive patient prognosis (Begley, 2009; Rosenvinge & Starke, 2005).

The large amounts of water consumed by the comparison group, which was not expected, further reiterates the need for inclusion of patients, caregivers and nurses in education about what the dysphagia management entails. Over and above the medical and hydration benefits of using water, its ability to quench one’s thirst adds to it being more acceptable and less aversive than a thickened liquid by patients with dysphagia (Colodny, 2005; Logemann *et al*., 2008). The current study confirms the benefit of water use in dysphagia intervention (i.e. for assessment and management). Simply explained, the human body is composed of approximately 60% of water. Any water that is consumed is absorbed by aquaporin channels within the lung where the blood vessels redistribute the water into the blood stream (Effros, Jacobs, Schapira & Biller, 2000; Panther, 2005). Hence the need for caution to be heeded in how water is used and under what oral hygiene circumstances (free of bacteria), as was seen in the different outcomes for the study and comparison group participants.

The use of videofluroscopy for participants in the study group, and barium swallow for four participants in the comparison group was noted as a limitation of the study. Having experience working within a government hospital, the researcher was aware that: (1) it was impossible to rely on equipment being in working order at any time and (2) patient waiting lists added to the delay in a procedure being conducted. For the purpose of the study, and with knowledge that the site had access to only barium swallow, videofluroscopy, shown to be more advantageous than barium swallow (Rofes *et al*., 2010), was selected as the imaging study of choice at a different hospital to that where the study was conducted. This procedure had no bearing on the intervention provided to the patients as it was conducted at the end of the intervention period. Despite the benefits that would have been gained conducting a videofluroscopy pre-intervention and post-intervention, the intent of the study was to conduct the oral care regimen in a typical acute government context. For this reason, the use of bedside evaluations was the primary dysphagia tool.

Studies have shown that in contexts where equipment and access to resources are limited, bedside assessments are still able to provide a clinician with valuable information about swallowing and what may be going wrong. Whilst lacking objective information to support what the clinician may be observing (Singh & Hamdy, 2006), a bedside evaluation and management offers the clinician immediate feedback and allows the clinician to implement changes and modifications to the course of management as necessary (McCullough & Martino, 2013).

## Conclusion

It is possible to reduce adverse medical effects of aspiration including fatality by implementing a cost-effective and low-resource oral care protocol for patients with dysphagia. An oral care and water provision regimen in addition to increasing patient compliance with dysphagia recommendations successfully prevents occurrence of aspiration pneumonia and prevents instances of aspiration from developing into aspiration pneumonia. This is something that holds potential value for resource constrained acute medical contexts, worldwide. The results seen amongst the eight patients from the study group who were aspirating during the course of intervention, but who did not develop aspiration pneumonia, confirms that it is possible to prevent the occurrence of aspiration pneumonia amongst patients who are aspirating, as long as oral care is implemented, the oral cavity remains clean when the patient consumes water and the oral cavity is cleaned after meals, to prevent aspiration of pieces of bolus residue within the oral cavity when the patient may be sleeping, for instance. The findings concur with those of El-Solh *et al*. (2003), who note that a lack of bacteria in the oral cavity prevents the build-up of infection in the lungs. Hence, as is possible in a chronic care context, reducing occurrence of aspiration pneumonia in an acute care context can also be attained (Yoneyama *et al*., 2002).

The oral care regimen tested in the current study was able to take cognisance of time and resources available to nurses working within an acute government hospital context. The added ‘limitation’ of the study was the regular monitoring of the nurses that occurred during the data collection. Thus it must be queried whether the oral cavity and water provision would have proceeded as it did, had the monitoring not occurred. Not documented in the study but which the findings of the study by Seedat (2013) showed, was that nurses requested more monitoring of the routines they conducted with patients. Nurses felt that this showed that their supervisors were interested and invested in what they were doing. Lack of monitoring meant that nobody was interested in whether a routine was completed or not, which they noted was particularly true for oral care. Hence, whilst regular monitoring of all nurses performing mouth care routines may not be a feasible notion within an acute government hospital context, the study does confirm that given the benefits associated with oral care, there needs to be a higher priority placed on the need for this routine to be done and monitored from time to time. As patient input/output details are recorded within files, formal recording of mouth care, with random checks, may offer an answer.

Despite the limitations of the study, which have already been acknowledged, some implications were identified. There is a growing need for more information dissemination amongst nurses. Similarly, patients and caregivers need to be more involved in decisions being made about their swallowing difficulty. Relevant professionals (for example nurses and speech-language pathologists) need to be better informed on aspects relating to the pathophysiological underpinning of aspiration pneumonia, consequences of aspiration pneumonia, symptomatology and the role of oral care in this process (Evans, 2001; Seedat, 2013). To enhance service delivery in dysphagia, improved inter-professional collaboration and information sharing with dentistry and respiratory specialists also holds value. Linked to this is the value of advocating the importance of inter-professional management of dysphagia at an undergraduate level and promoting inter-professional training, skill refinement and knowledge sharing in dysphagia.

As South Africa is a developing country, South African health professionals have varying levels of access to journals, the Internet and electronic support groups, which makes carry-over of this information to relevant health professionals challenging. Hence, national and provincial forums offer an ideal platform for such a protocol to be shared amongst clinicians.

The current study confirmed the combined benefit of oral care as well as free water provision to two specific target populations (i.e. patients with stroke and patients with traumatic brain injury, who presented with oropharyngeal dysphagia). The outcome of this schedule of oral care and water provision, and the oral care protocol itself, have not yet been proven for other populations, who may require additional considerations and modifications to be made. For example, head and neck cancer post-surgical patients, patients with dementia, patients with degenerative neurological conditions where muscle fatigue must be considered and patients with underlying respiratory conditions such as chronic obstructive pulmonary disease.
